# Iterative reconstruction of a global metabolic model of *Acinetobacter baylyi *ADP1 using high-throughput growth phenotype and gene essentiality data

**DOI:** 10.1186/1752-0509-2-85

**Published:** 2008-10-07

**Authors:** Maxime Durot, François Le Fèvre, Véronique de Berardinis, Annett Kreimeyer, David Vallenet, Cyril Combe, Serge Smidtas, Marcel Salanoubat, Jean Weissenbach, Vincent Schachter

**Affiliations:** 1 Genoscope (Commissariat à l'Energie Atomique) and UMR 8030 CNRS-Genoscope-Université d'Evry, 2 rue Gaston Crémieux, CP5706, 91057 Evry, Cedex, France

## Abstract

**Background:**

Genome-scale metabolic models are powerful tools to study global properties of metabolic networks. They provide a way to integrate various types of biological information in a single framework, providing a structured representation of available knowledge on the metabolism of the respective species.

**Results:**

We reconstructed a constraint-based metabolic model of *Acinetobacter baylyi *ADP1, a soil bacterium of interest for environmental and biotechnological applications with large-spectrum biodegradation capabilities. Following initial reconstruction from genome annotation and the literature, we iteratively refined the model by comparing its predictions with the results of large-scale experiments: (1) high-throughput growth phenotypes of the wild-type strain on 190 distinct environments, (2) genome-wide gene essentialities from a knockout mutant library, and (3) large-scale growth phenotypes of all mutant strains on 8 minimal media. Out of 1412 predictions, 1262 were initially consistent with our experimental observations. Inconsistencies were systematically examined, leading in 65 cases to model corrections. The predictions of the final version of the model, which included three rounds of refinements, are consistent with the experimental results for (1) 91% of the wild-type growth phenotypes, (2) 94% of the gene essentiality results, and (3) 94% of the mutant growth phenotypes. To facilitate the exploitation of the metabolic model, we provide a web interface allowing online predictions and visualization of results on metabolic maps.

**Conclusion:**

The iterative reconstruction procedure led to significant model improvements, showing that genome-wide mutant phenotypes on several media can significantly facilitate the transition from genome annotation to a high-quality model.

## Background

The diversity of bacterial metabolism and the perspective of engineering applications has spurred a steep increase in both the number of sequencing projects and the volume of high throughput experiments on bacteria. The need to interpret and integrate these datasets at the systems level has triggered the development of model-based computational methods [1]. Among them, the constraint-based modeling approach (CBM) has proved to be particularly efficient at integrating large-scale *omics *datasets related to metabolism, such as growth phenotypes, metabolite concentrations, or reaction fluxes [2]. In addition to providing a structured summary of metabolism-related knowledge for a given species, a constraint-based model allows the prediction and analysis of a variety of properties resulting from topological, stoichiometric, and physiological constraints known to apply at steady-state to its global metabolic network. Applications range from studies on evolutionary or physiological properties to the design of metabolic engineering strategies for biotechnological or therapeutical purposes [3]. Nearly twenty such models have been built so far [2], typically through extensive curation work, and, for some of them, through iterative refinement processes where models were progressively improved by comparison with experimental datasets [4].

Systematic evaluation of gene essentiality has proved to be a valuable resource for investigating gene functions; knockout mutant collections have been recently built in this aim for a number of bacteria [5-8]. Rigorous analysis of their results remains a challenging task, however, as gene essentiality depends on the environmental condition and the link between genes and essential functions may be blurred by genetic or metabolic redundancy [9,10]. Genome-scale metabolic models provide a valuable framework to help interpret essentiality screens, since they both recapitulate knowledge on metabolic networks and allow prediction of gene essentiality under well-defined conditions. They have also allowed meaningful cross-validation of reconstructed metabolic networks with sets of gene essentiality results, providing insights on potential erroneous or incomplete metabolic knowledge, and on possible improvements [4,11,12]. In this article, we systematically exploit inconsistencies between model predictions and experimental results to improve a metabolic model reconstruction.

Our focus is on *Acinetobacter baylyi *ADP1, a strictly aerobic γ-proteobacterium. Although phylogenetically close to the *Acinetobacter baumanii *pathogenic strains, responsible for a growing number of nosocomial infections [13], *A. baylyi *ADP1 is an innocuous soil bacterium. Because of its metabolic versatility and high competency for natural genetic transformation, it is a model organism of choice for genetic and metabolic investigations [14-16]. As a soil bacterium, *A. baylyi *is able to degrade a wide range of molecules, including components of suberin, a protective polymer produced by plants in response to stress. Its harmlessness, nutritional versatility, and high capacity for adaptation have led bacteria of the *Acinetobacter *genus to be used for a variety of biotechnological applications–including the degradation of pollutants (e.g. biphenyl, phenol, benzoate, crude oil, nitriles) and the production of valuable biochemical products such as lipases, proteases, bioemulsifiers, cyanophycine and different kinds of biopolymers [17,18]. Following its sequencing and expert annotation [19], a genome-wide single-knockout mutant library was generated (ADP1 mutant collection [8]), enabling the high-throughput assessment of mutant phenotypes in defined growth conditions.

We report below on the reconstruction and refinement of a genome-scale metabolic model for *A. baylyi *with the help of high-throughput experimental data. Following an initial reconstruction using metabolic information extracted from the genome annotation and the literature, the model was iteratively assessed and improved by comparing its predictions with (1) large-scale growth phenotyping results of the wild-type strain on 190 distinct environments, (2) genome-wide gene essentiality data from the mutant collection, and (3) conditional gene essentiality data derived from growth phenotyping of *A. baylyi *mutants on eight defined media. We examined each inconsistency between experimental results and model predictions, and corrected the model when sufficient justifying evidence could be collected. Combining the three refinement steps, 1262 out of 1412 predictions were initially consistent with experimental results. Among the inconsistent cases, 65 led to improvements, increasing the completeness and accuracy of the model. The final version of the model, called iAbaylyi^v4^, predicted accurately (1) 91% of the wild-type growth phenotypes, (2) 94% of the genome-wide gene essentialities, and (3) 94% of the phenotypic profiles of *A. baylyi *mutants on the tested media.

We developed a web interface which provides easy access to both model and experimental data. The interface allows browsing of the metabolic network, online computation of phenotype predictions, and comparison of predictions with experimental results [20].

## Results and discussion

### Initial model reconstruction

**Figure 1:**
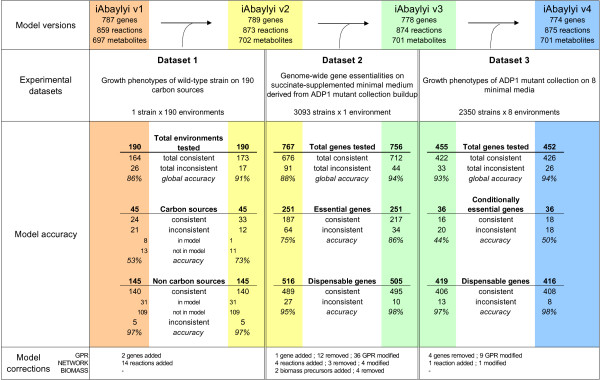
***A. baylyi *metabolic model refinement process**. *A. baylyi *metabolic model was iteratively refined in three steps using datasets of experimental results. The initial reconstruction iAbaylyi^v1 ^was assessed and improved using dataset 1; the resulting model iAbaylyi^v2 ^was then assessed and refined using dataset 2, yielding iAbaylyi^v3 ^which was again evaluated and refined using dataset 3, leading to the final model iAbaylyi^v4^. Since only mutants corresponding to dispensable genes in dataset 2 could be phenotyped in dataset 3, gene essentialities revealed in dataset 3 are medium-specific, i.e. conditionally essential. Genes classified as conditionally essential in dataset 3 are conditionally essential on at least one environment. Genes classified as dispensable are dispensable on all tested environments. Model accuracy figures indicates for each dataset and its corresponding models the counts of consistent and inconsistent predictions. Accuracy is computed as the fraction of consistent predictions among all predictions. For dataset 1, Biolog results for metabolites that were not in the model were counted as consistent with predictions if the metabolite was not a carbon source and inconsistent if the metabolite was a carbon source. Model corrections figures summarize the corrections performed on each model component.

**Figure 2:**
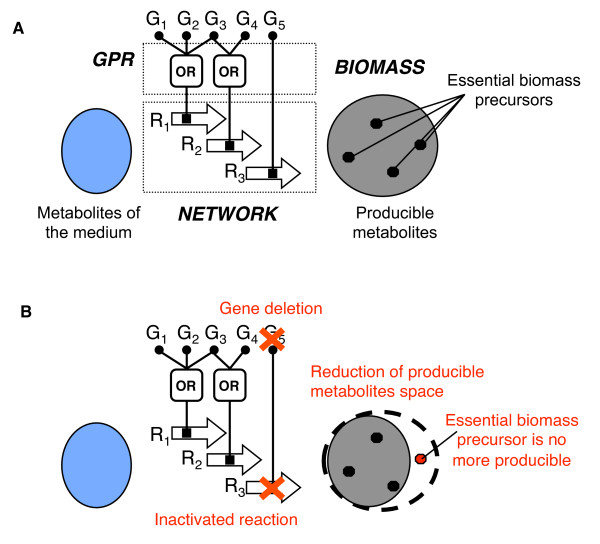
**Modeling framework**. (**A**) A metabolic model is represented as a combination of three model components: GPR Boolean rules associate genes (G_1 _to G_5_) with reactions (R_1 _to R_3_), the network of reactions defines the set of feasible biochemical transformations (illustrated by the arrows), and the set of essential biomass precursors defines the requirements for growth. Growth phenotypes are predicted by assessing whether all biomass precursors can be produced by the metabolic network from the set of metabolites from the medium [26] (see Methods) (**B**) Gene deletions potentially inactivate reactions, which in turn may reduce the space of producible metabolites. In case where a biomass precursor is no more producible, gene deletion is predicted lethal on the given medium.

The genome scale model of *A. baylyi *was iteratively reconstructed following a process depicted in Figure 1. We first built an initial draft model iAbaylyi^v1 ^using information from the genome annotation, metabolic pathways databases, and the literature. Although facilitated by the automated network reconstruction software PathoLogic [21], this initial reconstruction still required extensive manual curation (see Methods). The draft metabolic network generated by PathoLogic was first inspected to filter out and correct wrongly predicted pathways and reactions, and then completed by reviewing the expert genome annotations and the metabolic information contained in the literature. For instance, specific efforts were dedicated to properly include pathways accounting for the particular degradation capabilities of *A. baylyi*. Physiological information on *A. baylyi *was especially helpful to build the set of transport processes, as substrate specificities of transporters are difficult to deduce from genome annotation only. For each metabolite shown to be consumed by *A. baylyi *we added a corresponding transport reaction to the model. Out of 133 transporters, 23 were initially included in the model using this type of evidence only. The dependency between genes and reactions was modeled using Boolean rules, known as GPR (Gene-Protein-Reaction associations) [22]. These rules encode the presence of isozymes or enzymatic complexes for the catalysis of reactions, and predict the effect of genetic perturbations on the activity of reactions. GPR rules were first derived using homology with *E. coli *enzyme complexes [23] and then completed by manual curation. In order to model the metabolic and energetic demands associated with growth, we introduced a set of intermediary biomass reactions that synthesize generic cell constituents (e.g. protein, DNA, RNA, or lipid) from precursor metabolites, and a global growth reaction consuming them in proportion defined by studies of biomass composition [24,25]. Energetic parameters required to predict quantitative growth rate using Flux Balance Analysis (FBA) were assumed to be similar to those of *E. coli *model (see Methods)[22]. No accurate measurement of *A. baylyi *growth yields could be used to validate these parameters, however. While such validation would be required to get more accurate predictions of growth yields, the current parameters already provide good approximate values (see Additional file 1 for a sensitivity analysis on these parameters). For the purpose of qualitatively predicting growth ability using Metabolite Producibility analysis (see Figure 2) [26], we designed a reduced list of biomass precursors which are all essential for growth in *in vitro *conditions. We used this list to predict qualitative growth phenotypes and compare them with those of phenotyping experiments on *in vitro *environments. *In vivo *environments may impose harsher conditions requiring additional metabolic responses; this list therefore represents a minimal set of essential precursors that may need to be expanded to properly predict growth phenotypes on more realistic environments [27]. The Methods section provides more details on the reconstruction process.

**Figure 3:**
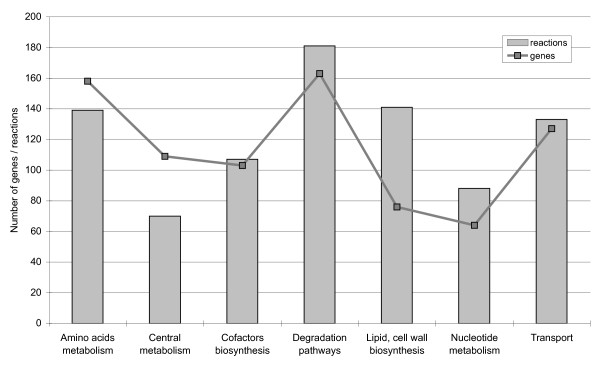
**Number of reactions and genes in iAbaylyi^v1 ^distributed by model metabolic categories**. Reactions were associated with a unique metabolic category. Genes linked to several reactions may be associated with multiple categories.

This initial reconstruction process led to the model iAbaylyi^v1 ^gathering 859 reactions grouped in 7 metabolic categories and 697 distinct metabolites, 109 of which could be transported from the environment. As depicted in Figure 3, the model accounts for all main processes of *A. baylyi *metabolism, including biosynthetic routes, energy metabolism, and catabolic pathways. Genomic islands of catabolic diversity endow *A. baylyi *with the ability to degrade a wide variety of soil compounds [19]. The metabolic model reflects this nutritional versatility, as 20% of its reactions are dedicated to the catabolism of external compounds. A list of specific compounds that can be degraded by *A. baylyi *is provided in Table 1.

**Table 1:** Some substrates involved in *A. baylyi *degradation pathways

Anthranillate	Octane
Benzoate	Straight chain dicarboxylic acids
Salicylate	Straight chain fatty acids
Catechol	Sarcosine
Chlorogenate	Propanaldoxime
Quinate	Propanenitrile
Shikimate	Propanamide
Coumarate	Malonate
Ferulate	Glucarate
Vanillate	Galactarate
Caffeate	Ethanesulfonate
Protocatechuate	

iAbaylyi^v1 ^involves 787 genes out of the 1518 confirmed or putative enzymatic and transport genes of *A. baylyi*. A large majority (94%, 681/726) of the enzymatic reactions (excluding transporters) were associated with at least one gene, while the lower proportion (83%, 110/133) of transport reactions linked to genes is explained by the extensive use of physiological data to include them. The association of nearly all reactions with a gene confers a high reliability to the model. The few reactions that were introduced with no associated gene are most often supported by indirect evidence and introduced in order to fill gaps (See Additional file 2).

**Figure 4:**
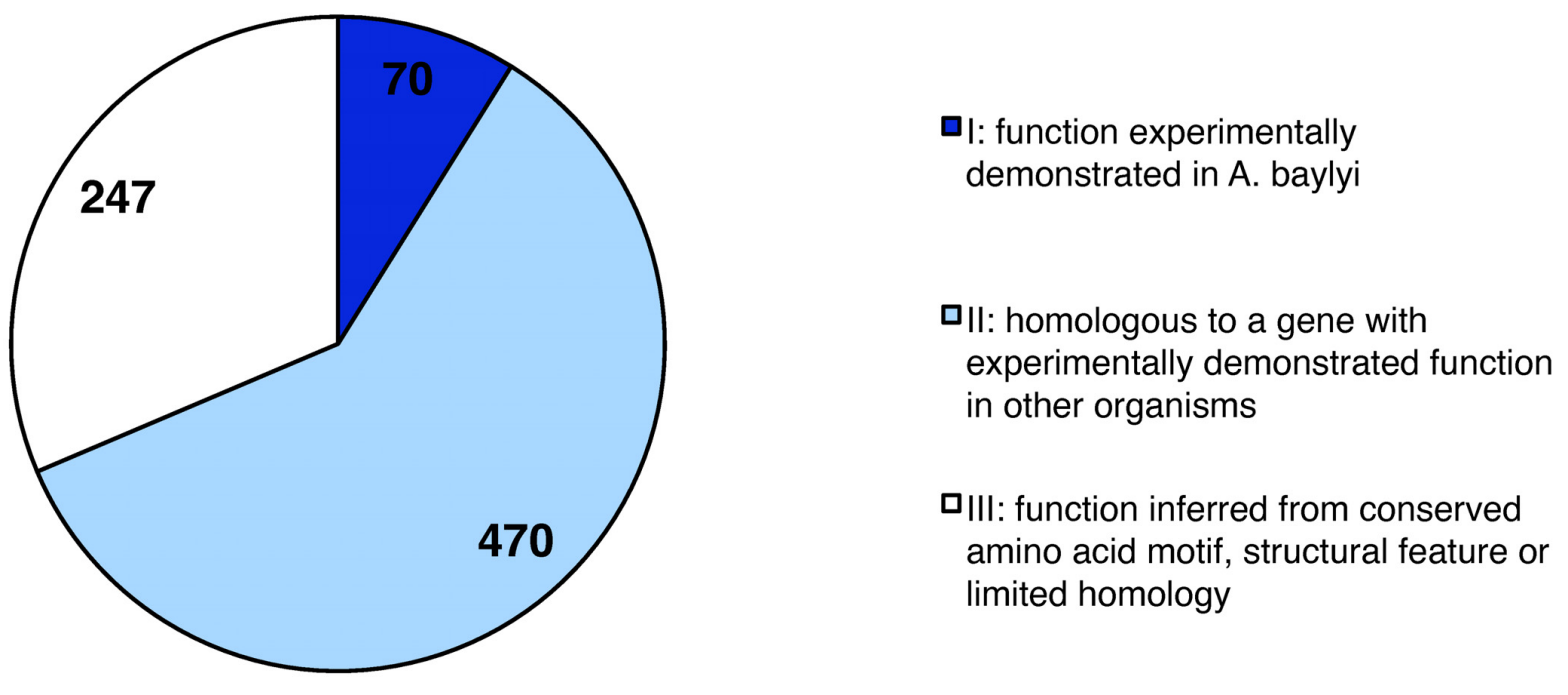
**Distribution of annotation confidence levels for genes included in iAbaylyi^v1 ^model**. Confidence levels were assigned according to the type of evidence supporting gene annotation.

Most *A. baylyi *genes were annotated by expert curation; a third of the model genes relied on evidence conferring them a medium confidence level, e.g. limited homology with genes of known function, or conservation of amino acid motifs (Figure 4). While the evidence for these genes does not fully prove the existence of associated enzymatic activities, it suggests them with sufficient strength to justify adding the corresponding reactions in the model. The level of evidence of each gene was tracked for later use in interpreting inconsistent behaviors. Out of 262 reactions to which these genes contribute, 85 are solely catalyzed by medium-confidence genes, some of these being essential to the model viability. In addition, 35% of all coding sequences are still of unknown function in *A. baylyi*, and may leave gaps in the actual metabolic network. Integration of additional experimental data was thus crucial in order to validate the metabolic network and correct it when necessary.

### Model validation and expansion using growth phenotype results

We used results of large-scale growth phenotyping experiments to perform a first round of model assessment and refinement. Using Biolog assays, we experimentally tested the wild-type strain ability to use 190 distinct metabolites as sole carbon and energy sources (see Methods). Using the model, we predicted the growth phenotypes of the wild-type strain on the corresponding *in silico *media and compared them to the experimental results.

Out of the 190 screened metabolites, 45 were found to be carbon and energy sources for *A. baylyi*. This relatively small fraction of carbon sources can be explained by the fact that Biolog microplates are only partially adapted to *A. baylyi*'s biotope: they feature sugars, nucleosides or amino acids but relatively few chemicals originating from plant compounds. iAbaylyi^v1 ^model predicted 24 of them and missed 21 (see Figure 1). Eight of the missed carbon source metabolites were already present in the model, but with no associated transporter. Amongst them, seven would also be predicted as carbon and energy sources had the corresponding transporters been included. In order to resolve these inconsistencies, we added for each of them a generic transport reaction accounting for *A. baylyi*'s ability to utilize these compounds (see Table 2). Growth on the remaining metabolite (2-ketobutyrate) was contradicted by an additional individual growth experiment.

**Table 2:** Biolog carbon sources inconsistently predicted by iAbaylyi^v1 ^and corresponding corrections

**Unpredicted Biolog carbon sources**	**21**
Prediction corrected by addition of transporter	7
3-ketobutyrate	
butyrate	
D-aspartate	
L-asparagine	
L-glutamine	
propionate	
pyruvate	
Prediction corrected by addition of degradation pathway	2
sorbate	
tricarballylate	
Biolog result contradicted by additional experiment	5
2-ketobutyrate	
alpha-D-glucose	
D-malate	
D-xylose	
L-arabinose	
Uncorrected inconsistencies – no relevant pathway found	7
2-hydroxybutyrate	
bromo-succinate	
D-lactate methyl ester	
methylpyruvate	
tween 20	
tween 40	
tween 80	
**Unpredicted Biolog non carbon sources**	**5**

Biolog result contradicted by additional experiment	1
4-hydroxybenzoate *	
Uncorrected inconsistencies	4
D-fructose	
D-serine	
L-arginine	
L-ornithine	

* result from [15]. Numbers provide the count of inconsistencies pertaining to each category.

Thirteen carbon source metabolites were unknown to the metabolic model. For two of them, sorbate and tricarballylate, we were able to identify degradation pathways and add them to the model (see Table 2). Sorbate, an unsaturated fatty acid, can be degraded by fatty acids oxidation enzymes, which were already included in the model for the degradation of other fatty acids. Sorbate transport and degradation reactions were therefore added to the model using the same set of genes. Recently, genes coding for tricarballylate transport (*tcuC*), oxidation to cis-aconitate (*tcuA *and *tcuB*), and for a regulatory protein required for *tcuABC *expression (*tcuR*) were identified in *Salmonella enterica *[28,29]. Highly homologous genes could be found in synteny in *A. baylyi*: ACIAD1536 (*tcuB*, 59% identity), ACIAD1537 (*tcuA*, 76% identity), ACIAD1541 (*tcuC*, 64% identity), ACIAD1539 (*tcuR*, 46% identity), and ACIAD1543 (*tcuR*, 44% identity). Following these clues, we expanded the model by implementing the corresponding transporter and degradation reaction, and annotated the corresponding genes. In four cases, dedicated growth experiments contradicted the Biolog result, weakening the case for further study (see Table 2). Finally, no relevant pathway could be found for the remaining seven unmodeled carbon sources. Further investigations are needed to identify the metabolic processes allowing *A. baylyi *to exploit these metabolites.

Conversely, only five of the 145 non-carbon source metabolites were wrongly predicted to be carbon sources by the model: 4-hydroxybenzoate, D-fructose, L-arginine, L-ornithine, and D-serine (see Figure 1 and Table 2). Experiments from [15] contradicted the Biolog result on 4-hydroxybenzoate, while additional individual experiments confirmed the Biolog results of the other four.

**Figure 5:**
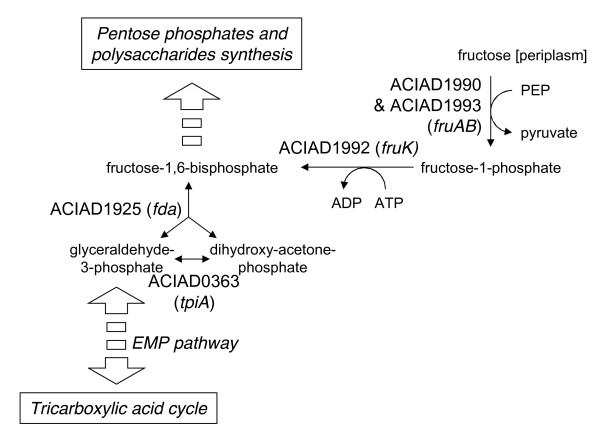
**Map of fructose utilization pathway in *A. baylyi***. Fructose utilization pathway produces fructose-1,6-biphosphate which should be a precursor for the biosynthesis of pentose phosphates and polysaccharides and for the tricarboxylic acid cycle. Model accordingly predicts growth with fructose as sole carbon source. Phenotyping experiments show no growth of *A. baylyi *with fructose as sole carbon source. Supposedly, the Embden-Meyerhof-Parnas (EMP) pathway may not operate in the glycolytic direction in *A. baylyi*, as already observed for glucose utilization [19,30]. See main text for details.

Interestingly, *A. baylyi *annotation describes a complete phosphotransferase (PTS) transport system for fructose (ACIAD1990 and ACIAD1993, *fruA *&*fruB*) coupled with a 1-phosphofructokinase (ACIAD1992, *fruK*) leading to fructose-1,6-bisphosphate (see Figure 5). In accordance with the annotation, the model predicts that fructose should be a carbon and energy source, yet this is not observed experimentally. To confirm the ability of the PTS system to transport fructose, we assessed experimentally the growth phenotype of the fructose bisphosphate aldolase (ACIAD1925, *fda*) knockout mutant (see Figure 5). The ΔACIAD1925 mutant could not be obtained on succinate-supplemented minimal media, reflecting the fact that Fda is required in the gluconeogenesis pathway to provide fructose-1,6-bisphosphate, an essential intermediate for building pentose-phosphates and polysaccharides. The mutant could however be obtained by adding fructose in the medium, showing that fructose could be imported into the cell and converted to fructose-1,6-bisphosphate. The reason why *A. baylyi *is unable to use fructose as a sole carbon source remains yet to be investigated. Hypothetically, *A. baylyi *may be unable to use the Embden-Meyerhof-Parnas (EMP) pathway in the glycolytic direction, as it has been observed for the dissimilation of glucose [19,30].

As is the case in *E. coli*, L-ornithine and L-arginine are degraded by *A. baylyi *using the arginine succinyltransferase (AST) pathway. This pathway allows *E. coli *to use them as nitrogen sources, but not as carbon sources. Putative explanations include unsuitable regulation and inadequate transport [31]. Similar reasons may explain *A. baylyi*'s inability to use L-ornithine and L-arginine as carbon sources.

*A. baylyi*'s genome annotation includes genes for D-serine transport (ACIAD0118 and ACIAD2662, *cycA*) and D-serine deaminase activity (ACIAD1048 *dsdA*), which should allow it to use D-serine as a carbon and nitrogen source. The interpretation of this inconsistency is also unclear; a similar unexplained inconsistency was pointed out in a study involving a metabolic model of *B. subtilis *[4].

Improvements to the model resulted in iAbaylyi^v2^, raising predictive accuracy on Biolog-measured phenotypes from 86% to 91% of the growth phenotypes (see Figure 1). Detailed results of the comparison with Biolog results can be found in Additional file 3.

### Systematic model improvement using gene essentiality data

In steps 2 and 3 of the model refinement process, we assessed and improved the model by comparing its predictions to experimentally determined gene essentialities, derived from the ADP1 mutant collection [8] (see Figure 1). Growth phenotypes of all single gene deletion mutants on the corresponding environments were predicted using metabolite producibility analysis (see Figure 2 and Methods). Predicted phenotypes were then compared to the genome-wide gene essentiality results in order to assess the accuracy of the model and to identify inconsistent predictions. Inconsistencies could be either *false essential *(genes falsely predicted essential by the model) or *false dispensable *(genes falsely predicted dispensable by the model) predictions. Since these inconsistencies are as many clues that the understanding of *A. baylyi*'s metabolism represented in the model is erroneous or incomplete, we examined them carefully in order to find interpretations and, when needed, refine the model.

We classified refinements into three categories according to the model component that was modified: GPR, NETWORK or BIOMASS (see Figure 2). These three components model different kinds of biological processes which contribute to determining the growth phenotype of mutant strains (see Methods). The GPR component, consisting of the GPR Boolean rules, computes the effect of the genetic perturbation on the activity of reactions in the model. The NETWORK component, the actual network of reactions, models the metabolic conversion capabilities of the organism. Finally, the BIOMASS component, consisting of the list of metabolites required for growth, models the biomass precursor requirements of the organism.

#### Model refinements

**Figure 6:**
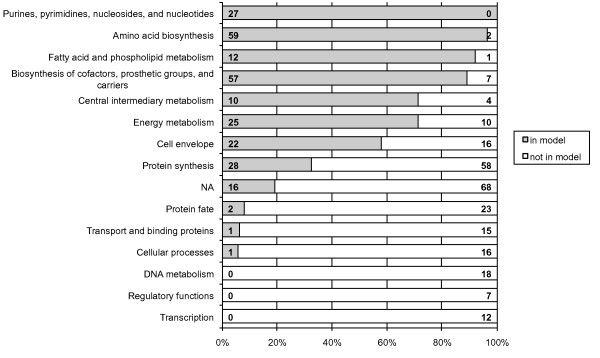
**Proportion of *A. baylyi *essential genes covered by iAbaylyi^v2 ^model distributed by TIGR role categories**. TIGR role categories were obtained from TIGR automated annotation of *A. baylyi *[67]. Some genes were associated with multiple functional classes. NA: no TIGR role has been assigned. For each role category, absolute numbers of genes in the model (left) and not in the model (right) are provided.

**Figure 7:**
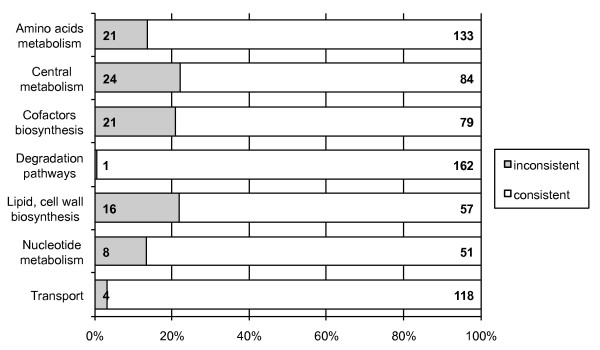
**Consistency of gene essentiality predictions for dataset 2 and iAbaylyi^v2 ^distributed by model metabolic categories**. Proportions of genes having inconsistent predictions for essentiality on succinate-supplemented minimal medium in iAbaylyi^v2 ^are shown for each model metabolic category. Genes linked to several reactions may be associated with multiple categories. For each metabolic category, absolute numbers of inconsistent (left) and consistent (right) gene essentiality predictions are provided.

We performed two iterations of refinement using gene essentiality data (see Figure 1). In a first step, we used gene essentialities established during the construction of the ADP1 mutant library to derive an intermediary version of the model iAbaylyi^v3^. This experimental dataset is nearly exhaustive as it covers 97% of all *A. baylyi *genes [8]. The mutant collection, built on succinate-supplemented minimal medium, revealed 499 essential genes for this medium. Half of these genes were present in the model (251/499), which is a significantly higher fraction than for all *A. baylyi *genes (24%, 789/3288). Although purely metabolic, the model thus already captured a large part of the bacterium's essential processes. The thoroughly curated but also purely metabolic *E. coli *model iAF1260 includes a similar proportion of *E. coli *essential genes on glucose-supplemented minimal medium (57%, 238/419) [12]. As shown in Figure 6, essential genes absent from the model were mainly related to functional categories lying outside of model scope, such as protein fate, DNA metabolism, transcription, or regulatory functions. On the other hand, essential genes involved in metabolic processes were largely covered by the model. iAbaylyi^v2 ^already showed good agreement with the observed gene essentialities as 88% of the predictions were identical to the experimental results (respectively 95% of dispensable genes and 75% of essential genes present in the model, see Figure 1). As depicted in Figure 7, inconsistencies were homogeneously distributed across the metabolic categories of the model, with an exception for Transport and Degradation pathways, which gathered few inconsistencies. Genes in these categories are typically dedicated to the use of external substrates and most of them are not required for growth on succinate medium only. Their metabolic role could thus not be evaluated in this first experiment: most were accordingly both observed and predicted as dispensable. Gene essentiality experiments on a variety of media were needed to assess the functions of these genes in the appropriate environmental context.

It is worth noticing that inconsistency results support our choice to include medium-confidence genes into the model. Genes associated with medium-confidence metabolic annotations did not trigger more inconsistencies then high-confidence level genes. 18% (47/268) of reactions including at least one medium-confidence gene in their GPR are associated with an inconsistent gene, a similar proportion to that of reactions containing only high confidence genes (14%, 75/527). We examined the 91 inconsistent predictions of this step and refined the model for 47 of them (see Table 3 and below for details on the corrections). The refinements were implemented in iAbaylyi^v3^, increasing global accuracy from 88% to 94%. Improvement was most noticeable for essential genes, as 86% were correctly predicted by iAbaylyi^v3^. As discussed below, a high number of false isozymes, triggering *false dispensable *predictions, were detected in this refinement step.

**Table 3:** Inconsistent gene essentiality predictions identified in refinement steps 2 and 3 and corresponding corrections and interpretations

**CORRECTION**			**56**	**NO CORRECTION**			**68**
**BIOMASS**			**10**	**Validated explanation**			**6**
	
biomass precursor not essential			9	experimental error			1
ACIAD0076 (rmlB)	D	step 2		ACIAD0108 (lldD)	D	step 3	
ACIAD0078 (rmlD)	D	step 2		known gap in the understanding of pathway			4
ACIAD0079 (rmlA)	D	step 2		ACIAD0856 (bioA)	E	step 2	
ACIAD0080 (rmlC)	D	step 2		ACIAD0857 (bioF)	E	step 2	
ACIAD0086 (epsM)	D	step 2		ACIAD0859 (bioD)	E	step 2	
ACIAD0099 (galU)	D	step 2		ACIAD2045 (bioB)	E	step 2	
ACIAD0101 (pgi)	D	step 2		unmodeled auxotrophy			1
ACIAD0104 (manB)	D	step 2		ACIAD3523 (metE)	E	step 2	
ACIAD2429 (cyoE)	D	step 2		** Hypothetical explanation **			** 32 **
				
missing essential biomass precursor			1	ACIAD0178 (atpI)	E	step 2	
ACIAD1374 (ispU)	E	step 2		ACIAD0180 (atpB)	E	step 2	
**GPR**			**34**	ACIAD0182 (atpE)	E	step 2	
				
activity simultaneously requiring all genes			3	ACIAD0183 (atpF)	E	step 2	
ACIAD0661 (hisG)	E	step 2		ACIAD0184 (atpH)	E	step 2	
ACIAD1257 (hisZ)	E	step 2		ACIAD0185 (atpA)	E	step 2	
ACIAD3103 (ilvH)	E	step 2		ACIAD0186 (atpG)	E	step 2	
gene associated to another essential reaction			1	ACIAD0187 (atpD)	E	step 2	
ACIAD2606	E	step 2		ACIAD0188 (atpC)	E	step 2	
isozyme not functional			22	ACIAD0556 (ndk)	D	step 2	
ACIAD0151 (guaA)	E	step 2		ACIAD0650 (argJ)	E	step 2	
ACIAD0249 (ribC)	E	step 2		ACIAD1150 (pyrC)	E	step 2	
ACIAD0871 (fabG)	E	step 2		ACIAD1346 (sodB)	E	step 2	
ACIAD1069 (lysS)	E	step 2		ACIAD1358 (rpiA)	E	step 2	
ACIAD1255 (epd)	E	step 2		ACIAD2282 (sahH)	D	step 2	
ACIAD1323 (purF)	E	step 2		ACIAD2314 (metZ)	E	step 2	
ACIAD1375 (cdsA)	E	step 2		ACIAD2458 (glnA)	E	step 2	
ACIAD1736 (accC)	E	step 2		ACIAD2842 (pckG)	E	step 2	
ACIAD1737 (accB)	E	step 2		ACIAD2847 (folD)	E	step 2	
ACIAD1925 (fda)	E	step 2		ACIAD3155 (mdh)	E	step 2	
ACIAD2227 (dctA)	E	step 2		ACIAD3349 (gltD)	E	step 2	
ACIAD2565 (gap)	E	step 2		ACIAD3350 (gltB)	E	step 2	
ACIAD2666	E	step 2		ACIAD3470 (msuE)	E	step 2	
ACIAD2907 (prs)	E	step 2		ACIAD3506 (aceF)	E	step 2	
ACIAD3062 (folK)	E	step 2		ACIAD0101 (pgi)	E	step 3	
ACIAD3249 (ribA)	E	step 2		ACIAD0546	E	step 3	
ACIAD3365 (murE)	E	step 2		ACIAD0556 (ndk)	D	step 3	
ACIAD3371 (gltX)	E	step 2		ACIAD1021	D	step 3	
ACIAD1710 (pcaC)	E	step 3		ACIAD1707 (pcaB)	E	step 3	
ACIAD2018 (ald1)	E	step 3		ACIAD1711 (pcaH)	E	step 3	
ACIAD2088 (aspQ)	E	step 3		ACIAD1712 (pcaG)	E	step 3	
ACIAD2983 (gcd)	E	step 3		ACIAD1744 (aspA)	E	step 3	
presence of an alternate enzyme			6	**No precise interpretation**			**30**
				
ACIAD1231 (argD)	D	step 2		ACIAD0072 (ugd)	E	step 2	
ACIAD1642 (uppP)	D	step 2		ACIAD0173 (rhtB)	E	step 2	
ACIAD2968 (ispA)	D	step 2		ACIAD0382 (ubiB)	D	step 2	
ACIAD1020 (acoL)	D	step 3		ACIAD0505 (purU1)	E	step 2	
ACIAD1715 (quiX)	D	step 3		ACIAD1482 (kdsD)	D	step 2	
ACIAD2984	D	step 3		ACIAD1483 (kdsC)	D	step 2	
spontaneously occurring reaction			1	ACIAD2283 (metF)	D	step 2	
ACIAD2819	D	step 3		ACIAD2290 (cydA)	E	step 2	
wrong complex subunit			1	ACIAD2525	E	step 2	
ACIAD0799	D	step 2		ACIAD2667 (pdxB)	D	step 2	
**NETWORK**			**12**	ACIAD2788	E	step 2	
				
false alternate pathway in the model			7	ACIAD2880 (sdhA)	D	step 2	
ACIAD0239 (ppa)	E	step 2		ACIAD2911 (panD)	D	step 2	
ACIAD0547 (proA)	E	step 2		ACIAD3503 (guaB)	E	step 2	
ACIAD1105 (adk)	E	step 2		ACIAD3510 (lpxC)	D	step 2	
ACIAD1920 (glnS)	E	step 2		ACIAD0086 (epsM)	E	step 3	
ACIAD2560 (proB)	E	step 2		ACIAD0335 (fadB)	E	step 3	
ACIAD3032 (proC)	E	step 2		ACIAD0382 (ubiB)	D	step 3	
ACIAD0901 (dut)	E	step 2		ACIAD0922	E	step 3	
missing alternate pathway in the model			5	ACIAD2070 (metI)	E	step 3	
ACIAD0106 (lldP)	D	step 2		ACIAD2282 (sahH)	D	step 3	
ACIAD0451 (katA)	D	step 2		ACIAD2283 (metF)	D	step 3	
ACIAD0930 (glpK)	D	step 2		ACIAD2667 (pdxB)	D	step 3	
ACIAD1045 (metH)	D	step 2		ACIAD2755	E	step 3	
ACIAD0106 (lldP)	D	step 3		ACIAD2875 (sucB)	E	step 3	
				ACIAD2876 (sucA)	E	step 3	
				ACIAD2880 (sdhA)	D	step 3	
				ACIAD2911 (panD)	E	step 3	
				ACIAD3071 (cysM)	E	step 3	
				ACIAD3549 (gshA)	E	step 3	

Inconsistencies identified during the refinement steps using mutant library essentialities (step 2) and mutant growth phenotypes on 8 media (step 3). Inconsistencies leading to corrections (left column) are listed according to the model component that was corrected: GPR, NETWORK, and BIOMASS. Inconsistencies with no correction (right column) are listed according to the level of interpretation that could be drawn. Numbers provide the count of inconsistencies pertaining to each correction or interpretation category. For each inconsistency, E or D indicates the experimental phenotype of the mutant: E: gene is essential (on at least one medium for step 3), D gene is dispensable (on all media for step 3).

In a second step, the model was evaluated against growth phenotyping assays of mutants from the ADP1 collection on 8 minimal media supplemented with varying carbon and nitrogen sources (see Table 4 and Methods). Since all *A. baylyi *mutants were first obtained on a succinate-supplemented minimal medium, essentialities revealed by these assays were strictly conditional. Furthermore, as the succinate-supplemented medium was already minimal, the set of conditionally essential genes was restricted to the genes directly related to the use of the tested carbon and nitrogen sources. These were chosen to involve different parts of *A. baylyi *secondary metabolism (see Table 4). Overall, 455 knockout mutants corresponding to genes in the model could be phenotyped (see Figure 1).

**Table 4:** Mutant phenotyping experiments: growth media and experimental results for genes included in iAbaylyi^v3^

**Source of^1^**		**Essentiality**		**Specific metabolic pathways *a priori *involved**
		
**Carbon**	**nitrogen**	**E**	**D**	
		
*acetate*	ammonia	5	431	Glyoxylate shunt
*L-asparagine*	ammonia	3	445	Asparagine and aspartate degradation
*D-2,3-butanediol*	ammonia	10	433	Butanediol to acetoin to acetyl-coa degradation, glyoxylate shunt
*D-glucarate*	ammonia	5	413	Glucarate to 2-oxoglutarate degradation
*β-D-glucose*	ammonia	7	432	Entner-Doudoroff pathway
*L-lactate*	ammonia	2	445	Lactate dehydrogenase
*quinate*	ammonia	8	436	Quinate to protocatechuate to acetyl-coa and succinyl-coa degradation
succinate	*urea*	3	442	Urease

^1 ^*Italic text *indicates the changed carbon or nitrogen source with respect to the medium used for mutant construction (succinate and ammonia).

E: number of conditionally essential genes

D: number of dispensable genes

Phenotyping experiments pointed out 2 to 10 conditionally essential genes (from the set of model genes) on each medium (Table 4). While a majority of these genes were essential on a single medium, some were found conditionally essential on several media. This revealed interdependencies between environments and might be related to processes specific to groups of environments. For instance, growth phenotypes on 2,3-butanediol and acetate exhibit similar characteristics since 2,3-butanediol is converted to acetate for its utilization [8]. The use of acetate as a carbon source requires the activation of the glyoxylate shunt, catalyzed by ACIAD1084 (isocitrate lyase) and ACIAD2335 (malate synthase G). These genes were therefore found to be essential on 2,3-butanediol and acetate only. Accordingly, the metabolic model correctly predicted the required use of this pathway and the subsequent essentiality of these genes on these media. As shown in Figure 1, iAbaylyi^v3 ^accurately predicted the phenotypic profiles of 93% of all genes, leaving 33 genes with inconsistent predictions on at least one medium. Nine of them led to model corrections, again mainly in the GPR component of the model (see Table 3). These corrections, implemented in iAbaylyi^v4^, slightly improved the predictive accuracy for mutant phenotypes (94%) while keeping the predictive accuracy for the previous datasets unchanged.

Combining both refinement steps, 56 out of 124 inconsistencies led to model corrections. In the following sections, we will discuss these gene essentiality inconsistencies in more details irrespective to the dataset that triggered them (see also Table 3). Model corrections will be presented according to the model component that was modified.

#### GPR corrections

**Figure 8:**
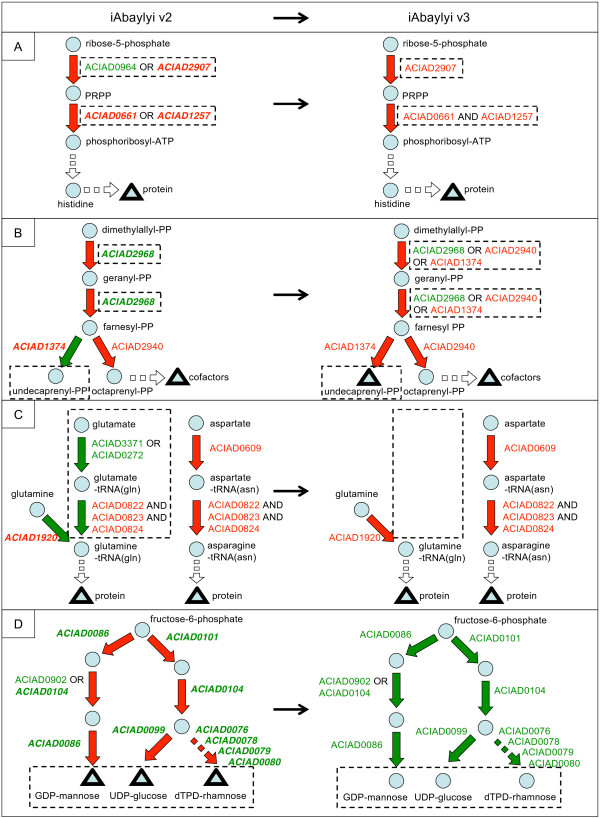
**Model correction examples**. Examples of model corrections performed between iAbaylyi^v2 ^(left) and iAbaylyi^v3 ^(right) models. Metabolites are depicted by blue circles and triangles, triangles indicating essential biomass precursors. Reactions are represented by arrows colored in red if they are predicted essential and in green if they are predicted dispensable. Gene names are indicated next to reaction arrows; they are written in red if they are experimentally essential and in green if they are dispensable. Genes with inconsistent predictions are written in bold italic. Dashed boxes indicate components that have been modified. Further evidence for model corrections are shown in main text and Additional file 3. (**A**) First steps of histidine biosynthesis. Unpredicted essentiality of ACIAD2907 encoding for ribose-phosphate diphosphokinase activity was corrected by removing the alternate gene ACIAD0964 from the reaction GPR. Unpredicted essentialities of ACIAD0661 and ACIAD1257, catalyzing the ATP phosphoribosyltransferase reaction, were corrected by assigning them as complex subunits instead of isozymes in the reaction GPR. (**B**) Isoprenoids biosynthesis. Unpredicted dispensability of ACIAD2968, catalyzing farnesyl-diphosphate and geranyl-diphosphate synthases activities, was corrected by adding ACIAD1374 (undecaprenyl-diphosphate synthase) and ACIAD2940 (octaprenyl-diphosphate synthase) as isozymes. Unpredicted essentiality of ACIAD1374 was resolved by adding undecaprenyl-PP to the set of essential biomass precursors. (**C**) Synthesis of charged glutamine-tRNA(gln) and asparagine-tRNA(asn). Unpredicted essentiality of ACIAD1920, encoding for glutaminyl-tRNA synthetase activity, was corrected by removing from the model the alternate pathway using aspartyl/glutamyl-tRNA amidotransferase enzyme (ACIAD0822-0824). (**D**) Biosynthesis of polysaccharides. Unpredicted dispensabilities of all genes involved in GDP-mannose, UDP-glucose, and dTDP-rhamnose synthesis were corrected by removing these three metabolites from the list of essential biomass precursors.

A majority of the model improvements (34/56) were applied to the GPR component, with a clear bias towards false dispensable inconsistencies: 26 GPR corrections pertained to experimentally essential genes against only 8 to experimentally dispensable genes (see Table 3). This large set of false dispensable predictions includes two main inconsistency types. In 22 cases, isofunctional genes with annotations of medium confidence were in fact unable to replace the activity of their deleted isozymes. For instance, ACIAD0964 and ACIAD2907 (*prs*) were identified in the initial reconstruction as isozymes for the catalysis of the ribose-phosphate diphosphokinase activity, which is required for the biosynthesis of 5-phosphoribosylpyrophosphate (PRPP) (see Figure 8A). The association of both genes to the activity relied on homologies with previously annotated genes in other organisms. The expected and predicted dispensability of ACIAD2907 was yet contradicted by its experimental essentiality. Looking further into the annotation evidence, ACIAD0964 function was supported by only limited homologies to previously known genes (second best hit after ACIAD2907 with *E. coli *gene *prsA*, with 25% identity). Conversely, ACIAD2907 function was supported by a stronger homology with *E. coli *gene *prsA *(68% identity) whose ribose-phosphate diphosphokinase has been experimentally confirmed [32]. The combination of the observed gene essentialities with the limited homology supporting the annotation of ACIAD0964 led us to correct the model by removing ACIAD0964 from ribose-phosphate diphosphokinase GPR. On the other hand, the functions of some isozymes with medium confidence level were corroborated by the gene essentialities. For instance, two isozymes were indirectly confirmed to have a dihydroxy-acid dehydratase activity, which is essential for the synthesis of valine, leucine and isoleucine. Two duplicate genes were associated with this activity: ACIAD1266 (*ilvD*) and ACIAD3636. While the annotation of ACIAD1266 is supported by a strong homology with *E. coli *gene *ilvD *(74% identity) whose activity has been experimentally shown [33], ACIAD3636's function was supported only by weaker homologies with the reference genes (37% identity with *E. coli *gene *ilvD*). Gene knock-outs revealed that both genes were dispensable while the essentiality of other genes in the pathway strongly suggested that the dihydroxy-acid dehydratase activity was required. This result strongly suggests that both genes could back up each other and therefore indirectly corroborates the functional assignment to ACIAD3636.

Further examination revealed that the duplicate genes are also found together in other organisms, including *Bradyrhizobium japonicum *and *Bordetella bronchiseptica*, and that *S. cerevisiae *possesses the gene ILV3, with a confirmed activity [34], which is homologous to ACIAD3636 (51% identity). Overall, amongst the reactions which were essential to iAbaylyi^v2 ^viability and associated with an isozyme of medium confidence-level, 8 showed agreement between predictions and phenotypes while 11 triggered inconsistencies. In other words, while some medium-level genes were discarded thanks to essentiality data, a comparable fraction of genes was indirectly confirmed. This observation provides additional confirmation that essentiality data represents a valuable resource, as it helps validate or discard gene functions supported by reasonably good but non-conclusive evidence. It also provides an *a posteriori *validation of the usefulness of including medium-level annotations in the initial model, as failing to do so would have resulted in a significant loss of information in the *A. baylyi *metabolic model.

For three false dispensable predictions, we uncovered enzymatic complexes or functional dependencies between genes that were absent from the initial reconstruction: genes thought to be isozymes were in fact jointly required to catalyze the reactions. As an illustration, ACIAD0661(*hisG*) and ACIAD1257 (*hisZ*) were initially assigned as isozymes of ATP phosphoribosyltransferase reaction in the pathway of histidine biosynthesis (see Figure 8A). The observed essentiality of both genes suggested that they were both necessary to the activity. Further examination of the literature confirmed that, unlike in *E. coli*, ACIAD0661 forms a complex with ACIAD1257 [35]. In *E. coli, hisG *codes for an enzyme that is active on its own and is not part of a complex.

Amongst the false essential predictions which led to modifications of the GPR component, six cases involved associating additional enzymes to reactions. For instance, ACIAD2968 (*ispA*, farnesyl diphosphate synthase) was observed to be dispensable, even though it is the only catalyst of two reactions essential for the biosynthesis of isoprenoids, which are the precursors of vital cofactors (see Figure 8B). Previous work showed for *E. coli *that *ispA *was dispensable and that *ispB *(octaprenyl diphosphate synthase) and *ispU *(undecaprenyl diphosphate synthase) could perform these activities [36]. *A. baylyi*'s homologues to these genes – ACIAD2940 (*ispB*) and ACIAD1374 (*ispU*) – were therefore added as isozymes of ACIAD2968 for both reactions (see Figure 8B).

The remaining types of GPR refinement involved associating genes with already existing essential reactions (ACIAD2606: associated with nicotinate-nucleotide adenylyltransferase activity, which is essential for NAD biosynthesis), adding new complex subunits (ACIAD0799: falsely considered as a sulfite reductase subunit and replaced by ACIAD2981 after further investigations) or assigning spontaneous activity (ACIAD2819: encodes for gluconolactonase activity which has been shown to occur spontaneously [37]). See Additional file 3 for further details on these corrections.

#### NETWORK corrections

Twelve gene essentiality inconsistencies from datasets 2 and 3 led us to improve the NETWORK component of the model (see Table 3). Two types of inconsistencies fall within this category. On the one hand, false dispensable predictions may indicate that alternate pathways present in the model are either inactive for the experimental conditions under observation or not present at all. Seven discrepant predictions led us to reconsider alternate pathways in the model. For instance, ACIAD0822, ACIAD0823, and ACIAD0824 (*gatABC*), annotated as aspartyl/glutamyl-tRNA amidotransferase, catalyzed in iAbaylyi^v2 ^the synthesis of charged glutamine-tRNA and charged asparagine-tRNA through the transamidation of misacylated glutamate-tRNA(Gln) and aspartate-tRNA(Asn) (see Figure 8C). Charged glutamine-tRNA can also be produced by the direct charging of glutamine on its tRNA using the glutaminyl-tRNA synthetase enzyme (ACIAD1920, *glnS*), however. The observed essentiality of ACIAD1920 is inconsistent with the redundancy of these two pathways, suggesting that the transamidation of glutamate-tRNA(Gln) does not occur *in vivo*. Furthermore, aspartate-tRNA(asn) transamidation is actually the only way of producing asparagine, as *A. baylyi *is lacking both asparagine synthetase and asparaginyl-tRNA synthetase enzymes. This result strongly suggests that, in *A. baylyi*, ACIAD0822-0824 genes are predominantly employed for asparagine-tRNA synthesis. To account for ACIAD1920 essentiality, we thus removed the glutamate-tRNA(Gln) transamidation pathway from the metabolic network.

On the other hand, false essential predictions may suggest that alternate pathways are missing from the model. Corrections of this type involve searching for new metabolic activities, a task that is open-ended and exploratory in nature and is likely to require additional experimental work. Five inconsistencies led to the addition of new reactions to the model, mainly for the transport of metabolites.

#### BIOMASS corrections

Ten inconsistent gene essentiality predictions led to modifications of the BIOMASS component (see Table 3). False essential inconsistencies can reveal biomass precursors that are not necessary to the viability of the cell on the tested environments, yet commonly produced by the wild-type strain. For instance, a large fraction of the BIOMASS modifications (8/10) were found in the biosynthesis of polysaccharides. Based on studies of the lipopolysaccharides composition of *Acinetobacter *species [38,39], three nucleotide sugars were initially included in the list of essential biomass precursors. All genes specifically involved in the synthesis of these sugars were found to be dispensable for growth on these *in vitro *environments (see Figure 8D). Further investigations are needed to analyze the composition of polysaccharides in the corresponding mutants and interpret the robustness to these deletions. Although dispensable in our experimental growth conditions, complete polysaccharides are likely to be essential on more realistic environments. Cell surface polysaccharides play an important role to help colonization and prevent desiccation while secreted polysaccharides are assumed to provide *A. baylyi *with better uptake capabilities of hydrophobic compounds in natural environments [19,40]. In order to account for these viable phenotypes on our experimental conditions, all three sugars were removed from the list of biomass precursors.

Conversely, false dispensable inconsistencies may uncover essential metabolites that were initially overlooked. For instance, undecaprenyl diphosphate, a cofactor required for the synthesis of peptidoglycan, was not part of the biomass precursors list in iAbaylyi^v2^. ACIAD1374 (*ispU*, undecaprenyl pyrophosphate synthetase), involved in its synthesis, was observed essential, although predicted dispensable (see Figure 8B). As this cofactor is regenerated during the peptidoglycan building process, its synthesis was actually not required at steady state. We therefore added undecaprenyl diphosphate to the list of essential metabolites in order to account for its required synthesis and resolve the unpredicted essentiality of ACIAD1374. An alternate method was recently introduced to account for the non-constitutive requirement for cofactors [27]. Small consumption terms are added for each cofactor in the equation of reactions involving them, thereby creating a replenishing flux of cofactor when reactions are active. This replenishing flux enforces the synthesis of the cofactor when required. While this method allows discarding cofactors from the general biomass requirements, it involves remodeling the reaction equations in an artificial manner.

#### Interpretation of remaining inconsistencies

The analysis of inconsistent predictions did not always lead to model refinement. Either the explanation of the discrepancy did not lead to model refinement, or no explanation interpreting the discrepancy could be validated.

Six discrepancies were confidently interpreted yet did not lead to model modifications (see Table 3). In one case, we identified a wrong experimental result. Four inconsistencies pertained to the pathway of biotin synthesis, whose essentiality could not be accounted for by the model. Since the initial step of this pathway is unknown, it could not be linked to the metabolic network, preventing the model from simulating biotin synthesis. One inconsistency was caused by a requirement for a cofactor that could not be modeled. Two different methionine synthase enzymes catalyze the conversion of homocysteine to methionine: one B12-independent encoded by ACIAD3523 (*metE*) and one B12-dependent encoded by ACIAD1045 (*metH*). Since coenzyme-B12 is neither synthesized by *A. baylyi *nor provided in the experimental media, the ΔACIAD3523 mutant was unable to use the MetH enzyme to synthesize methionine. The model could not account for this B12 auxotrophy of the ΔACIAD3523 mutant. In order to properly account for the dependency between MetH activity and the presence of a cofactor, the replenishing flux method can be employed [27] or the modeling framework could be extended by introducing rules that state which conditions are required for the enzymes to be active. The introduction of this additional layer of rules has already been proposed to account for regulatory constraints [41] and may be helpful to explain a number of inconsistent phenotypes.

For 62 inconsistencies, we could not reach a validated explanation within the scope of this global analysis (see Table 3). For 32 of them, we could formulate hypothetical interpretations, all of which need experimental confirmation. A high proportion of these possible interpretations involve regulatory processes. For instance, *A. baylyi *possesses like *E. coli *two distinct enzymes for glutamate synthesis: glutamate synthase, encoded by ACIAD3350 (*gltB*) and ACIAD3349(*gltD*), and glutamate dehydrogenase, encoded by ACIAD1110 (*gdhA*). In *E. coli*, these pathways were shown to be regulated in response to nitrogen limitations [42]: glutamate synthase is used at low ammonium concentrations while glutamate dehydrogenase is used at high ammonium concentrations. *E. coli *strains lacking glutamate synthase show severe growth deficiency at low ammonium concentrations [42]. Similarly, ACIAD3350 and ACIAD3349 were found essential in *A. baylyi *on the succinate-supplemented minimal medium. These phenotypes contradicted model predictions, which considered the alternate pathway for glutamate synthesis. Further investigation would be required to fully understand the regulatory processes at work in this pathway for *A. baylyi *and extension of the modeling framework should be conducted to account for regulatory processes within the model.

The remaining 30 inconsistencies could not be given a clear interpretation and also require further investigations.

### The final model: iAbaylyi^v4^

The overall refinement process led to the final model iAbaylyi^v4 ^gathering 774 genes, 875 reactions and 701 metabolites (see Figure 1). iAbaylyi^v4 ^integrates all refinements resulting from the three experimental datasets introduced in this work. Accordingly, its predictions are consistent with the experimental results in 91% of the cases for dataset 1, 94% of the cases for dataset 2, and 94% of the cases for dataset 3. Compared with iAbaylyi^v1^, it was expanded by 19 reactions and 2 genes, while 3 reactions and 16 genes were removed in the refinement process (see Figure 1, Model corrections).

### An online software tool for the exploration of *Acinetobacter baylyi *metabolism

In order to facilitate the exploration of *A. baylyi *metabolism using the genome scale model, we created NemoStudio [20] (Combe *et al*, in preparation), a web interface combining a simulation layer for the model with AcinetoCyc, *A. baylyi *Pathway-Genome Database [21]. NemoStudio gathers data on functional genomics annotations, metabolic reactions and pathways, and experimental mutant phenotyping results within a single interface. Additionally, it allows performing phenotype predictions using the constraint-based model.

AcinetoCyc gathers information on the metabolic network of *A. baylyi *and is used to display interactive metabolic maps. After its initial automated construction using PathoLogic [21], AcinetoCyc has been undergoing constant curation. It includes all metabolic reactions present in the model.

**Figure 9:**
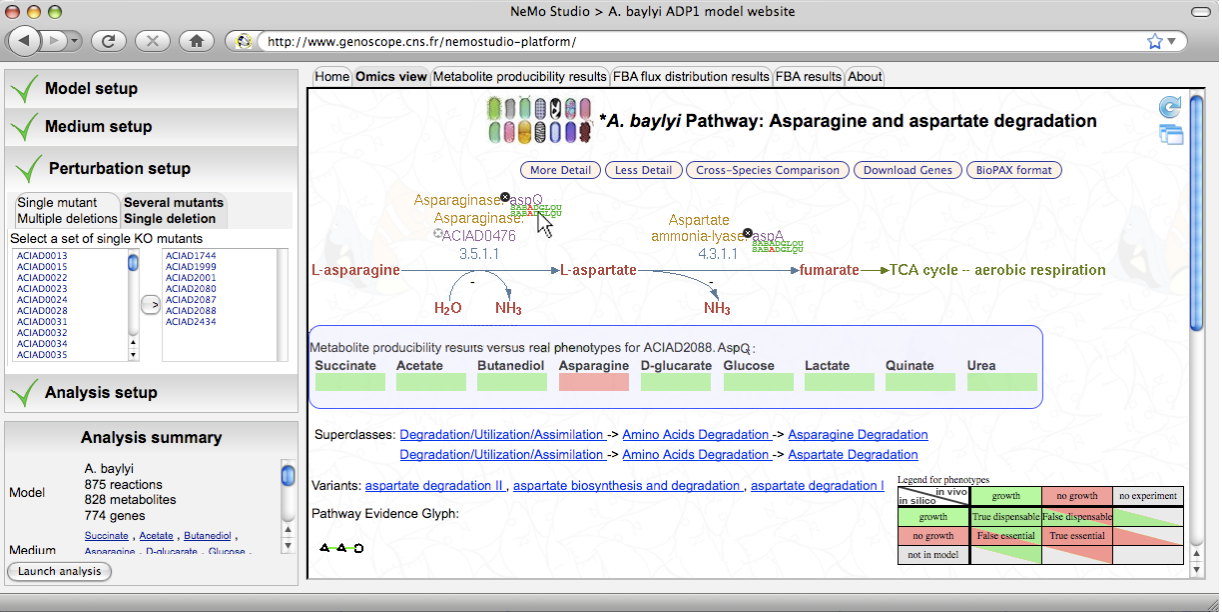
**Screenshot of NemoStudio web interface**. The web interface is divided in two parts. The left panel is dedicated to setting the analyses performed on the metabolic model. Simulated media, gene knockouts and type of analysis (metabolite producibility or flux balance analysis, see Methods) can be set in this panel. The right panel displays results in various formats for the selected type of analysis. The "omics view" part maps the predicted and experimental results on AcinetoCyc metabolic maps.

NemoStudio integrates the latest version of *A. baylyi *metabolic model, iAbaylyi^v4^. Growth phenotype predictions can be performed for any set of environmental conditions and genetic perturbations of this study. We implemented both Flux Balance Analysis (FBA) and Metabolite Producibility methods to predict growth phenotypes (see Methods). When performed on sets of environmental conditions and sets of gene deletions, prediction results are displayed in a table format in parallel to the actual experimental results. Predictions can thus be readily compared with the experimental observations. Furthermore, predicted and experimental phenotypes are both displayed on AcinetoCyc metabolic maps, and conversely gene deletions can be directly set from these metabolic maps (see Figure 9). When performed for a single environment and a single genetic perturbation, FBA predicts an optimal flux distribution towards biomass production; these fluxes are both displayed in a table and on AcinetoCyc metabolic pathways.

The availability of this resource as a web interface makes it easily usable by scientists interested in *A. baylyi *metabolism. Compared with previous web-based software for genome-scale metabolic modeling [27], the *A. baylyi *NemoStudio interface provides better interactivity, direct visualization of results on metabolic maps and integrated comparison with experimental data. By interfacing as much as possible results deriving from systems level analyses with experimental data of various forms, it allows the simultaneous exploitation of both information types.

## Conclusion

In this work, we reconstructed a genome-scale model of *Acinetobacter baylyi *metabolism from the annotation of its genome, metabolic knowledge reported in the literature, and results of high-throughput experiments. The model provides a curated and structured representation of this species's metabolism for use both as a reference and as a foundation for further study. The reconstruction accounts for 875 reactions, 701 distinct metabolites, and 774 genes, and includes nearly all metabolic routes and biochemical conversions identified for *A. baylyi*. A significant proportion of reactions belong to pathways of secondary metabolism that are characteristic of *A. baylyi*'s physiology and lifestyle. The model thus reflects the specific ability of *A. baylyi *to utilize various chemicals originating from plant metabolism, e.g. aromatic acids, hydroxylated aromatic acids, or straight chain dicarboxylic acids. It may assist or even drive future investigations on this bacterium, helping for instance interpret other types of experimental data beyond growth phenotypes, or engineer its metabolism. An increasing number of metabolic engineering strategies are being designed with the help of genome-scale metabolic model predictions [43,44]: the availability of the *A. baylyi *model should facilitate efforts towards biotechnology goals. The *A. baylyi *model may also serve as a basis for the reconstruction of metabolic models of the pathogen strains *Acinetobacter baumanii*. These strains, which are involved in serious nosocomial infections worldwide and have acquired multidrug-resistance capabilities[13], share a significant number of metabolic genes with *A. baylyi *[45]. This model is also the fourth genome-scale bacterial metabolic model to be accompanied by an exhaustive mutant library (with *E. coli *[5,12], *Bacillus subtilis *[4,6], and *Pseudomonas aeruginosa *PAO1 [46,47]). The proximity between *A. baylyi *and *P. aeruginosa*, and to a lesser extent *E. coli*, and the availability of model/mutant library pairs provides an invaluable setup for comparing the metabolism of different species [8].

Several rounds of comparisons of model predictions to large-scale experimental results led to significant model improvements. First, growth phenotypes of the wild-type strain on 190 distinct environments resulted in the addition of 9 transporters and 2 pathways to the model. After improvement, the model accounted correctly for the growth phenotypes on 173 of the 190 environments. Secondly, we assessed the model against gene essentiality results on 9 defined environments. In contrast with wild-type growth phenotypes, these data can bring indirect information on the gene functions or on the existence of alternate pathways. Investigation on the causes of inconsistencies led us to modify the model in 56 cases out of 124 inconsistent predictions. All model components were modified, the GPR component gathering most of the improvements. The model accuracy in predicting mutant growth phenotypes increased from 88% to 94% on succinate-supplemented minimal medium and from 93% to 94% for the combined conditional gene essentiality results on 8 media. High-throughput phenotype clearly improved the quality of the model and expanded our understanding of *A. baylyi *metabolism, providing a valuable complement to the annotation and the literature. The refinement process was particularly useful in validating or contradicting functional annotations that stood in the "grey zone", i.e. for which the annotation process provided only medium-level evidence.

Conversely, the model allowed systematic evaluation of the results of these high-throughput experiments by comparing them to its predictions. Inconsistencies directly targeted informative experimental results for which further investigation are required. As shown in this work, not all inconsistencies led to model improvements. Some of them could be interpreted in terms of biological processes lying outside the scope of the modeling framework, probably regulation in most cases. In addition, a significant number of discrepancies reported in this work remained unexplained or led to hypotheses in need of confirmation through further study.

The process described here was driven by expert curation: each inconsistency was manually examined in order to search for an interpretation and a possible model correction, a labor-intensive proposition. The systematic use of such experimental data for model refinements would be greatly facilitated by the development of computational methods assisting the curator with his task, however. A number of methods have been developed to search for variants of model which match better with additional experimental data, mainly by seeking additions or removals of reactions in the metabolic network [48,49]. These methods have already proven efficient at suggesting metabolic pathways that account for previously unexplained growth on specific environments [48]. While they can be adapted to handle growth phenotypes of knockout mutant strains, they do not involve the gene-reaction association component of the model, which is shown here to be the main area of model improvement. The association between genes and reactions can be complex as regulatory constraints may interfere with the actual gene function assignments. Computational strategies are therefore needed to help interpret the consequences of gene essentiality data on gene activities.

Deriving the full benefits from a metabolic model entail both accessing its components and using its predictive capabilities. We realized the former by providing access to a detailed metabolic pathways database, the latter through a software tool that performs online predictions, both being coupled at the level of genes and reactions and accessible through a single, highly-interactive interface. This interface allows end-users to carry systems level predictions, and compare them with corresponding experimental observations, putting the consequences of modeling in the context of the detailed biological information that went into the model. This tool should therefore provide researchers interested in *A. baylyi *metabolism with a valuable resource for investigating its phenotypic and physiological properties.

## Methods

### Initial reconstruction process

The initial reconstruction of the metabolic network was carried out using data provided by (i) the genome expert annotation [19], (ii) the BioCyc metabolic pathway database automatically generated from these annotations [21] and (iii) various literature resources on biochemistry, including textbooks, reviews and journal publications (see Additional file 2). The genome annotation was downloaded from the MaGe interface [50,51] and used as input of the Pathway Tools software [21] in order to generate a BioCyc automatic reconstruction of the metabolic network. The predicted pathways were classified into 7 metabolic categories (central metabolism, nucleotide metabolism, amino acids metabolism, lipid & cell wall metabolism, degradation pathways, cofactor biosynthesis, transport) and examined manually before being included in the model. In order to meet the requirements of the modeling framework the mass balance and reversibility of the reactions were checked.

Reversibility of the reactions was determined from literature evidence when available or based on simple thermodynamic considerations [52]. Proton translocation efficiencies of reactions of the respiratory chain were assumed to be similar to those of *E. coli *[53]. Resulting P/O ratio can range between 0.5 to 2, depending on the types of cytochrome oxidase and NADH dehydrogenase that are used. Reactions using generic compounds (for example a nitrile or a polymer of undetermined length) were instantiated with defined representative metabolites. In this respect, polymeric pathways were expanded into chains of specific reactions. Large polymeric molecules such as the acyl carrier protein (ACP) or tRNAs were included in the model when they were involved as substrate cofactors of biochemical reactions. Their specific synthesis was not considered in the model. Dependency between reactions and genes were coded by Gene-Protein-Reaction (GPR) Boolean relationships (see below). Using the Cyclone interface to BioCyc [54], we implemented a simple method based on gene homologies between *Escherichia coli *and *Acinetobacter baylyi *to infer enzyme complexes and find AND Boolean associations between genes. Information from the literature was used to close gaps in the metabolic pathways, include pathways specific to *A. baylyi *that were unknown to the metabolic databases, and check the predicted pathways, for instance for the specificity of the cofactors. Physiological information derived from the literature [15,55-59] was used together with genome annotation tools, e.g. TransportDB [60], to add transport reactions in the model. A generic transport reaction was added to the model for each metabolite shown to be utilized by *A. baylyi*. A fixed biomass composition was chosen according to data found in the literature for strains growing on standard media (see Additional file 4). This biomass composition was used to build the reduced list of essential biomass precursors and derive a biomass reaction for Flux Balance Analyses (see below). To help properly account for all metabolic requirements associated with growth, we decomposed the biomass reaction into a set of intermediary biomass reactions synthesizing generic cell constituents (e.g. protein, DNA, RNA, or lipid) from precursor metabolites and a global growth reaction consuming them according to the chosen biomass composition. See Additional file 4 for details on these reactions.

### Modeling framework

The metabolic model is composed of three components, namely GPR, NETWORK and BIOMASS. The GPR component models the dependency between genes and reactions using Boolean functions usually called gene-protein-reaction (GPR) associations [22]). For each reaction, a Boolean rule encodes how genes are related to the activity. Genes that are required together are linked with an AND relation while isofunctional genes are linked with an OR relation. The set of GPR associations yields the set of potentially active reactions given the set of available genes.

The NETWORK component models the metabolic network using the constraint-based modeling framework [3]. This framework describes the distributions of reaction fluxes that are compatible with constraints that derive from basic physical assumptions or specific biological information. They are usually formulated as linear constraints, which allow to explore the fluxes solution space using linear programming tools. The main constraint is imposed by the steady-state assumption, represented by the matrix equation:

*S*·*v *= 0

where *S *is the stoichiometric matrix of the metabolic network and *ν *the vector of reaction fluxes. The stoichiometric matrix is a matrix of size (*m *× *n*) where *m *is the number of metabolites and *n *the number of reactions. Each element *S*_*i*,*j *_of the matrix represents the relative stoichiometric coefficient of metabolite *i *in reaction *j*. Additional constraints on the fluxes, such as irreversibility and capacity constraints, are imposed by inequalities in the form:

*ν*_*lb, i *_≤ *ν*_*i *_≤ *ν*_*ub, i*_

where *ν*_*lb*,*i *_and *ν*_*ub*,*i *_are respectively the lower and upper bounds of the flux of reaction *i*.

Environmental conditions are applied to the model by constraining the exchange fluxes of extracellular metabolites. Exchange fluxes are sink reactions allowing to control the input or output of metabolites in the model. They are constrained to 0 ≤ *ν*_*i *_≤ ∞ for metabolites absent from the medium and -∞ ≤ *ν*_*i *_≤ ∞ for metabolites present in the medium, except for limiting nutrients for which a maximum uptake rate is chosen (-*ν*_*uptake *_≤ *ν*_*i *_≤ ∞). When simulating the metabolic network of a knockout mutant, the activity of each reaction is determined by evaluating its GPR association according to the set of removed genes. Fluxes of the inactivated reactions are constrained to be equal to zero.

The BIOMASS component models the essential metabolic requirements for growth. It consists of a list of metabolites that are considered to be essential biomass precursors. Growth phenotype is therefore determined by checking their producibility [26]. To do so, the steady-state constraints for the essential biomass precursors are changed to strict producibility constraints:

$$\{\begin{array}{l} S_{internal}\cdot \nu =0\\ S_{biomassprecursors}\cdot \nu \geq \varepsilon \\ \nu_{lb,i}\leq \nu_{i}\leq \nu_{ub,i} \end{array}$$

where *S*_*internal *_is the stoichiometric matrix without the biomass precursors, *S*_*biomass precursors *_the stoichiometric matrix restricted to the biomass precursors and *ε *a vector of small reals, taken as 10^-3^. Linear programming tools are used to query for a flux distribution fulfilling this set of constraints. If a flux distribution could be found, the model predicted growth, otherwise it predicted no growth.

In order to assess quantitative growth defects, Flux Balance Analyses (FBA) were performed [3]. A biomass reaction was introduced in the model to quantitatively account for the respective contributions of constituent metabolites in the biomass composition (see Additional file 4). Using linear programming, the flux through this reaction was maximized under all constraints, representing the maximal growth rate achievable by the model. Energetic parameters, including growth associated (GAM) and non growth associated (NGAM) maintenance fluxes, were assumed to be similar to those of *E. coli *model [22]. We chose to set NGAM to a constant ATP hydrolysis flux of 10 mmol/h/gDW and GAM to a value of 40 mmol/gDW of ATP in the growth reaction. In all simulations, upper bounds of nutrient exchange fluxes were set to 10 mmol/h/gDW for carbon sources and 100 mmol/h/gDW for other nutrients (see Additional file 2).

Model simulations were performed within FluxAnalyzer [61] and MATLAB^® ^(The MathWorks Inc., Natick, MA) using the YALMIP optimization toolbox [62] and MOSEK optimization solver (Mosek ApS, Copenhagen, Denmark).

### Availability of metabolic model

The metabolic model is available both as Excel and SBML files (see Additional files 2 and 5) and will be submitted to the Biomodels.net repository [63]. Whenever possible, cross-references for the model reactions and species to AcinetoCyc [20], KEGG [64] and BiGG [65] databases are provided.

The model is accessible through the NemoStudio web interface [20]. NemoStudio supports growth phenotype predictions, and comparison to experimental results, as well as browsing of model pathways through an interface with AcinetoCyc [20].

### Growth phenotyping of the wild-type strain

Growth phenotyping experiments of *A. baylyi *were performed by Biolog, Inc. (Hayward, CA) following experimental procedures described in [66]. Basically, growth of wild-type strains of *A. baylyi *was monitored in PM1 and PM2 microplates containing a defined minimal medium supplemented with 190 distinct carbon sources. The Biolog quantitative growth measures were discretized to yield growth/no-growth qualitative phenotypes by choosing thresholds based on the negative growth control measures and previously known growth phenotypes for *A. baylyi*. Growth phenotypes that were inconsistent with model predictions were checked by examining results from previous work [15], or retesting them individually. Detailed results of Biolog experiments are provided in Additional file 3.

### Growth phenotyping of the mutant strains

Detailed experimental protocol for the growth phenotyping of the mutant strains is described in [8]. Basically, using 96-wells plates, the mutant strains were grown in liquid MA minimal media (31 mM Na2HPO4, 25 mM KH2PO4, 18 mM NH4Cl, 41 μM nitrilotriacetic acid, 2 mM MgSO4, 0.45 mM CaCl2, 3 μM FeCl3, 1 μM MnCl2, 1 μM ZnCl2, 0.3 μM (CrCl3, H3BO3, CoCl2, CuCl2, NiCl2, Na2NoO4, Na2SeO3)) supplemented with 25 mM of carbon sources. Succinate/urea medium was composed of MA minimal medium without NH4Cl supplemented with 25 mM of succinate and 20 mM of urea. Absorbance at 600 nm of 24 h cultures was measured to monitor growth. Experiments were performed in duplicates. Measures with discrepant repeats or with weak precultures were discarded from the analyses. Repeats were filtered according to the following rule: a measure was kept if either (1) both repeats were under the growth threshold or (2) the relative difference between the repeats was lower than 50% of the highest value. A threshold of a tenth of the mean absorbance was chosen to classify the mutants in growth or no growth categories. This threshold was chosen particularly low in order to consider as essential only mutants with marked fitness defect.

## Authors' contributions

MD reconstructed the initial model, performed model predictions, interpreted inconsistent phenotypes, applied model corrections, and wrote the manuscript. FLF reconstructed the initial model and developed the NemoStudio software tool. VDB participated in the experimental phenotyping and the interpretation of inconsistent phenotypes. AK and DV participated in the initial reconstruction and the interpretation of inconsistent phenotypes. CC and SS developed the NemoStudio software tool. MS participated in the experimental phenotyping and the interpretation of inconsistent phenotypes. JW participated in the design and the coordination of the study. VS conceived of the study, participated in its design and coordination, and contributed to writing the manuscript. All authors read and approved the final manuscript.

## Supplementary Material

Additional file 1 **Sensitivity on GAM and NGAM parameters of growth rate predictions**. This file contains two plots showing the effect of changing growth associated (GAM) and non growth associated (NGAM) maintenance parameters on quantitative growth rate predictions with iAbaylyi^v4^. Click here for file

Additional file 2 **Genome-scale metabolic models**. This file contains the description of all model versions as well as information on reactions, species, biomass precursors, modeled environments and literature references used for the model reconstruction. Click here for file

Additional file 3 **Experimental data and model refinements**. This file gathers the experimental results used for model refinements, the model predictions, and the corrections/interpretations associated to the inconsistent predictions. Click here for file

Additional file 4 **Determination of biomass composition of *A. baylyi***. This file gathers all information used to reconstruct the biomass assembly reactions in the metabolic model. Click here for file

Additional file 5 **Genome-scale metabolic model in SBML format**. This file contains the latest model iAbaylyi^v4 ^in SBML format . Click here for file
