# Supplementary material for: Iterative reconstruction of a global metabolic model of Acinetobacter baylyi ADP1 using high-throughput growth phenotype and gene essentiality data
Source: BMC Syst Biol. 2008 Oct 7;2:85. doi: 10.1186/1752-0509-2-85 (PMC2606687; doi:10.1186/1752-0509-2-85)

Additional file 1 - Sensitivity of growth rate predictions on GAM and NGAM parameters

To evaluate the effect of growth associated (GAM) and non growth associated (NGAM) maintenance parameters on quantitative growth rate predictions, we predicted growth rates using FBA for GAM and NGAM values varying in a range of +/- 100% around their initial values (resp. 40 mmol ATP/gDW and 10 mmol ATP/h/gDW). We chose succinate-supplemented minimal media as the growth environment (see supplementary data 1). Succinate maximal uptake rate was set between 0 and 20 mmol/h/gDW.

1. Effect of GAM parameter on the predicted growth rate.
NGAM parameter was set to its initial value (10 mmol ATP/h/gDW).

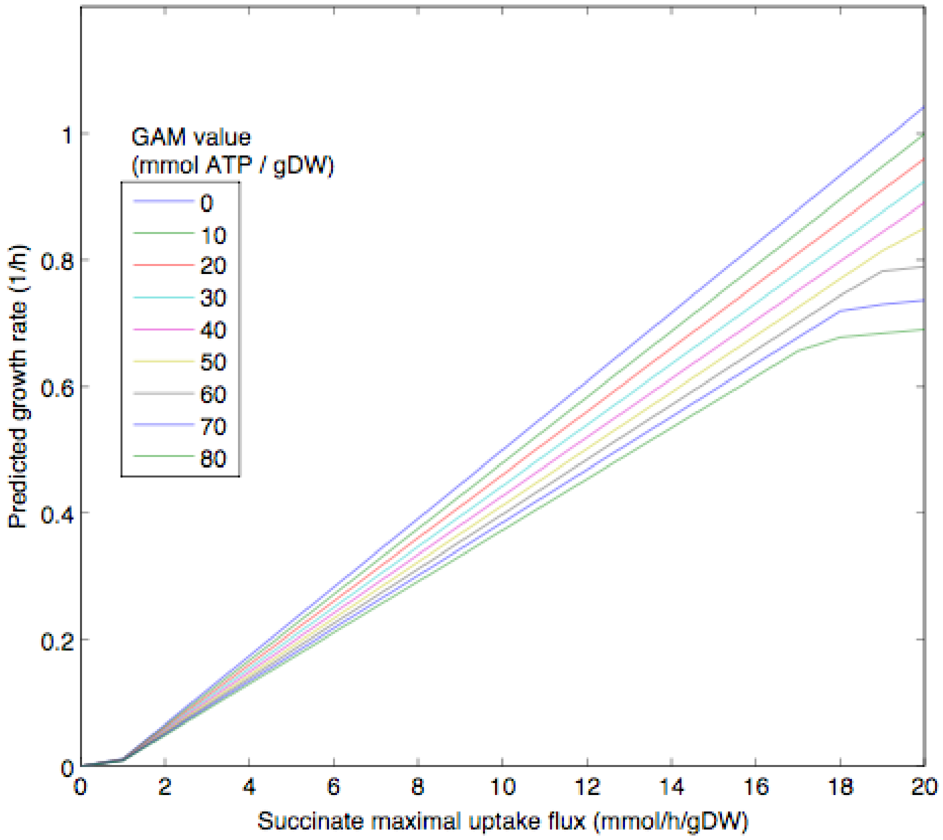

2. Effect of NGAM parameter on the predicted growth rate.
GAM parameter was set to its initial value (40 mmol ATP/gDW).

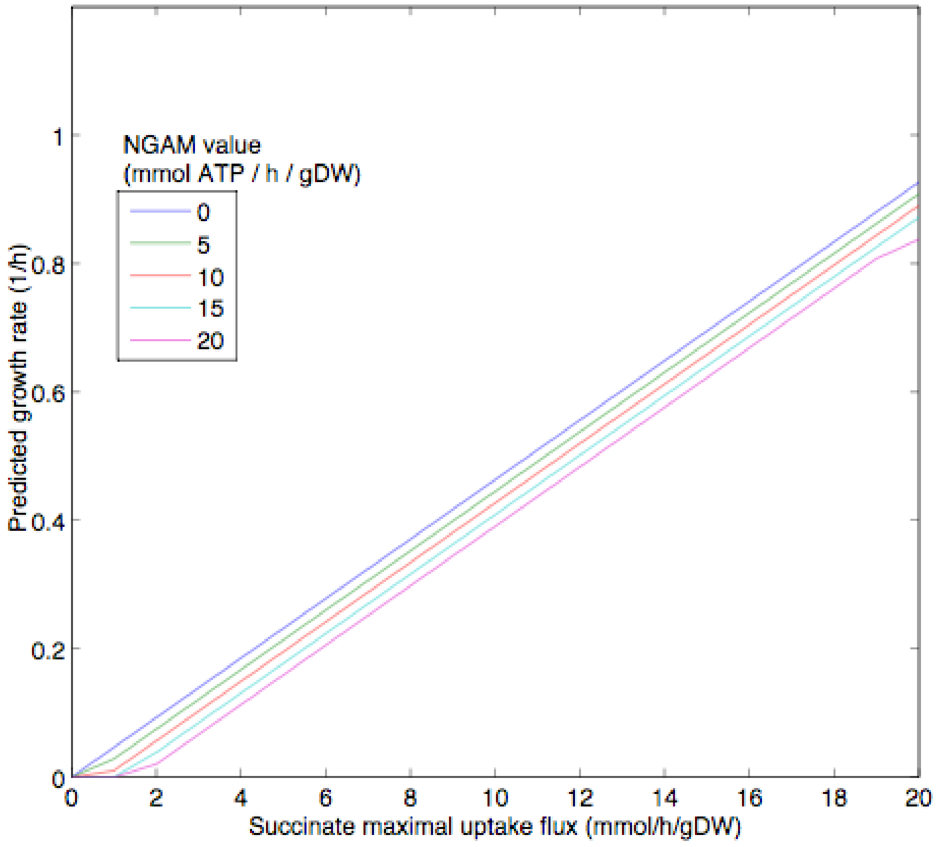

Supplement: Additional file 1 — Sensitivity on GAM and NGAM parameters of growth rate predictions. This file contains two plots showing the effect of changing growth associated (GAM) and non growth associated (NGAM) maintenance parameters on quantitative growth rate predictions with iAbaylyiv4. [file 1752-0509-2-85-S1.pdf]
